# Ensuring trustworthy use of artificial intelligence and big data analytics in health insurance

**DOI:** 10.2471/BLT.19.234732

**Published:** 2020-02-25

**Authors:** Calvin W L Ho, Joseph Ali, Karel Caals

**Affiliations:** a Faculty of Law, Cheng Yu Tung Tower, Centennial Campus, The University of Hong Kong, Pokfulam, Hong Kong Special Administrative Region, China.; b Department of International Health, Johns Hopkins Bloomberg School of Public Health, Baltimore, United States of America.; c Centre for Biomedical Ethics, Yong Loo Lin School of Medicine, National University of Singapore, Singapore.; Correspondence to Calvin W L Ho (email: cwlho@hku.hk).

## Abstract

Technological advances in big data (large amounts of highly varied data from many different sources that may be processed rapidly), data sciences and artificial intelligence can improve health-system functions and promote personalized care and public good. However, these technologies will not replace the fundamental components of the health system, such as ethical leadership and governance, or avoid the need for a robust ethical and regulatory environment. In this paper, we discuss what a robust ethical and regulatory environment might look like for big data analytics in health insurance, and describe examples of safeguards and participatory mechanisms that should be established. First, a clear and effective data governance framework is critical. Legal standards need to be enacted and insurers should be encouraged and given incentives to adopt a human-centred approach in the design and use of big data analytics and artificial intelligence. Second, a clear and accountable process is necessary to explain what information can be used and how it can be used. Third, people whose data may be used should be empowered through their active involvement in determining how their personal data may be managed and governed. Fourth, insurers and governance bodies, including regulators and policy-makers, need to work together to ensure that the big data analytics based on artificial intelligence that are developed are transparent and accurate. Unless an enabling ethical environment is in place, the use of such analytics will likely contribute to the proliferation of unconnected data systems, worsen existing inequalities, and erode trustworthiness and trust.

## Résumé

Les progrès technologiques en matière de big data (un terme qui désigne de grandes quantités de données extrêmement variées, provenant de différentes sources et pouvant être traitées rapidement), de sciences de l'information et d'intelligence artificielle peuvent améliorer le fonctionnement du système de santé, mais aussi promouvoir des soins personnalisés et servir l'intérêt public. Néanmoins, ces technologies ne permettront pas de remplacer les composantes fondamentales du système de santé, comme le leadership éthique et la bonne gouvernance, ni d'éviter la nécessité de créer un environnement déontologique et réglementaire solide. Le présent document se penche sur la définition de cet environnement déontologique et réglementaire solide pour l'analyse des big data dans le domaine de l'assurance maladie, et fournit à titre d'exemple les mécanismes de protection et de participation qu'il convient d'instaurer. En premier lieu, imposer un cadre de gouvernance précis et efficace est essentiel au traitement des données. Des normes juridiques doivent être promulguées, tandis que les assureurs doivent être encouragés et incités à adopter une approche centrée sur l'humain, tant dans leur conception que dans leur utilisation de l'analyse des big data et de l'intelligence artificielle. Deuxièmement, il faut mettre en place un processus clair et responsable afin d'expliquer quels types d'informations sont susceptibles d'être employés et à quelles fins. Troisièmement, les personnes concernées doivent avoir la possibilité de déterminer de quelle manière leurs données personnelles sont gérées et régies, en étant activement impliquées dans ce processus. Et quatrièmement, les assureurs et les organes de gouvernance, dont les régulateurs et législateurs, doivent collaborer pour faire en sorte que l'analyse des big data basée sur l'intelligence artificielle soit correcte et transparente. À moins d'établir un environnement éthique, l'usage d'une telle analyse entraînera probablement la prolifération de systèmes de données non connectés, l'aggravation des inégalités actuelles ainsi qu'une perte de confiance et de fiabilité.

## Resumen

Los avances tecnológicos relativos a los macrodatos (es decir, grandes cantidades de datos muy variados de muchas fuentes diversas que pueden procesarse rápidamente), las ciencias de los datos y la inteligencia artificial pueden mejorar las funciones del sistema sanitario y promover la atención personalizada y el bien público. No obstante, estas tecnologías no sustituirán los componentes fundamentales del sistema sanitario, como el liderazgo ético y la gobernanza, ni evitarán la necesidad de un entorno ético y normativo sólido. En el presente documento se examina cómo podría ser un entorno ético y normativo sólido para el análisis de macrodatos en el ámbito de los seguros médicos, y se describen ejemplos de mecanismos de protección y participación que deberían establecerse. En primer lugar, es fundamental contar con un marco claro y eficaz de gestión de datos. Es necesario promulgar normas jurídicas y alentar e incentivar a las aseguradoras para que adopten un enfoque centrado en el ser humano en el diseño y la aplicación de análisis de macrodatos e inteligencia artificial. En segundo lugar, es necesario un proceso claro y responsable para explicar cómo y qué información se puede utilizar. En tercer lugar, se debe facultar a las personas cuyos datos puedan ser utilizados mediante su participación activa en la determinación de cómo se pueden gestionar y regular sus datos personales. En cuarto lugar, las aseguradoras y los órganos de gobierno, incluidos los reguladores y los responsables de formular políticas, deben colaborar para garantizar que los análisis de macrodatos basados en la inteligencia artificial que se elaboren sean transparentes y precisos. A menos que exista un entorno ético adecuado, el uso de esos análisis probablemente contribuirá a la proliferación de sistemas de datos sin conexión, empeorará las desigualdades existentes y reducirá la fiabilidad y la confianza.

## ملخص

يمكن للتطورات التكنولوجية في البيانات الضخمة (وهي الكميات الكبيرة من البيانات شديدة التنوع من العديد من المصادر المختلفة والتي يمكن معالجتها بسرعة)، وعلوم البيانات والذكاء الاصطناعي، تحسين وظائف النظام الصحي وتعزيز الرعاية الشخصية والصالح العام. ومع ذلك، إلا أن هذه التقنيات لن تحل محل المكونات الأساسية للنظام الصحي، مثل القيادة والحوكمة الأخلاقية، أو تجنب الحاجة إلى بيئة أخلاقية وتنظيمية قوية. سوف نناقش في هذه الورقة كيف تبدو البيئة الأخلاقية التنظيمية القوية بالنسبة لتحليلات البيانات الضخمة في التأمين الصحي، كما نصف أمثلة للضمانات والآليات المشتركة التي يجب إنشاؤها. أولاً، يعد وجود إطار حوكمة واضح وفعال للبيانات أمراً حيوياً. يجب تفعيل المعايير القانونية كما يجب تشجيع جهات التأمين ومنحها الحوافز لاعتماد نهج يركز على الإنسان في التصميم واستخدام تحليلات البيانات الضخمة والذكاء الاصطناعي. ثانياً، من الضروري وجود عملية واضحة وخاضعة للمساءلة تهدف إلى شرح المعلومات التي يمكن استخدامها وكيف يمكن استخدامها. ثالثاً، يجب دعم الأشخاص الذين يمكن استخدام بياناتهم، وذلك من خلال انخراطهم بشكل نشط في تحديد كيفية إدارة بياناتهم الشخصية والتحكم فيها. رابعاً، تحتج جهات التأمين وهيئات الحوكمة، بما في ذلك الجهات التنظيمية وواضعي السياسات، إلى العمل معًا لضمان أن تكون تحليلات البيانات الضخمة القائمة على الذكاء الاصطناعي، والتي تم تطويرها، تتميز بالشفافية والدقة. ما لم تكن هناك بيئة أخلاقية مناسبة، فإن استخدام هذه التحليلات من المحتمل أن يسهم في انتشار أنظمة للبيانات غير المتصلة، ويزيد من سوء الحالات القائمة لعدم المساواة، ويحد من الثقة والجدارة بالثقة.

## 摘要

大数据（即，可以快速处理大量不同来源的高度差异化数据）、数据科学和人工智能领域的技术进步可以改善医疗系统功能，促进个性化护理和公益服务。然而，这些技术不会取代医疗系统中的道德领导和治理等基本组成要素，也不会消除对稳健的道德和监管环境的需求。在本文中，我们讨论了医疗保险大数据分析中的稳健道德和监管环境可能是什么样子的，并且举例描述了应该建立的保障和参与机制。首先，一个清晰有效的数据治理框架至关重要。需要制定法律标准，并且鼓励和激励保险公司在设计和使用大数据分析和人工智能方面秉承以人为本的理念。第二，必须有一个明确的问责流程来解释可以使用哪些信息以及如何使用这些信息。第三，对于数据被采用的人员，应该通过积极参与决定如何管理和治理其个人数据的方式为其赋权。第四，保险公司和治理机构，包括监管机构和政策制定者，需携手合作，确保基于人工智能开发的大数据分析是透明且准确的。除非具备有利的道德环境，否则使用此类分析很可能会导致未连接的数据系统的分散，加剧现有的不均衡情况，并降低可靠性和可信度。

## Резюме

Стремительное развитие технологий в области больших данных (то есть больших объемов разнородных данных из самых разных источников, которые можно быстро обрабатывать), обработки и анализа данных и искусственного интеллекта может совершенствовать функции систем здравоохранения и способствовать разработке индивидуального подхода к оказанию медицинской помощи и обеспечению благосостояния общества. Тем не менее использование этих технологий не сможет заменить фундаментальные компоненты системы здравоохранения, такие как этика руководства и управления, или устранить необходимость создания надежной этической и нормативной среды. В этой статье авторы обсуждают, как может выглядеть надежная этическая и нормативная среда для аналитики больших данных в сфере медицинского страхования, и рассматривают примеры защитных мер и механизмов участия, которые необходимо разработать. Во-первых, важнейшее значение имеет разработка четкой и эффективной системы управления данными. Необходимо принять правовые стандарты, которые будут создавать стимулы для страховщиков использовать подход, ориентированный на нужды людей, для разработки и использования аналитики больших данных и искусственного интеллекта. Во-вторых, необходимо разработать четкий и прозрачный метод определения типа данных, которые могут использоваться, а также способ использования таких данных. В-третьих, люди, чьи данные могут использоваться, должны наделяться полномочиями посредством активного участия в определении методов управления их личными данными. В-четвертых, страховщикам и органам управления, включая директивные органы и органы регулирования, необходимо сотрудничать для обеспечения прозрачности и точности аналитики больших данных, полученной на основе разработанного искусственного интеллекта. В отсутствие благоприятной этической среды использование такой аналитики будет скорее способствовать распространению несвязанных систем данных, усугублению существующего неравенства и подрыву авторитетности этой аналитики и доверия к ней.

## Introduction

Health insurance is widely recognized as crucial to advancing universal health coverage (UHC), which is a component of the sustainable development goals.^1^ Most research shows that health insurance coverage reduces the risk of death and improves health outcomes, with certain vulnerable populations, such as infants, people with disabilities and people living with the human immunodeficiency virus, benefitting more than the general population.^2^ In addition, evidence suggests that continuity of health insurance coverage, as opposed to sporadic or no coverage, is particularly effective in maintaining health.^2^ Different models of insurance schemes have been established by different countries based on their socioeconomic conditions and cultural contexts. There are three broad and often overlapping categories of health insurance schemes: national or social health insurance; voluntary and private health insurance; and community-based health insurance.^3^^,^^4^ These insurance schemes have different payment requirements, with many governments contributing to the schemes to ensure financial sustainability or to expand coverage to people who cannot afford to pay.^5^ As countries progress towards UHC, they will need to consider more effective means of raising sufficient funds to meet rising health-care costs, reduce reliance on direct payments to finance health-care services, and improve efficiency and equity in access to health care.^6^ In some countries, for example the United Kingdom of Great Britain and Northern Ireland, policies introduced to advance UHC have contributed to increasing emphasis on enhancing patient choice and provider competition.^7^^,^^8^

Excessively bureaucratic systems for health insurance can raise ethical, legal and social concerns related to interference with professional judgement and patient choice. Decisions on what services should be covered and how they can be sustained to meet the health needs of insured people also raise concerns.^3^^,^^9^^,^^10^ These concerns and the challenges they present are not new; they arise from difficult compromises that need to be made in decisions on risk distribution between parties that have potentially conflicting interests and unequal access to relevant information. This situation is referred to as problems of agency, which broadly refers to conflict of interest inherent in a relationship where one party is expected to act in the best interests of another. For example, the problem of moral hazard is an agency problem that arises when an insured person no longer has an incentive to guard against health risks because of actual or perceived transfer of financial risk from the insured person to the insurer.^11^^,^^12^ While cost sharing, that is, co-payments, co-insurance and deductibles, is widely used as a simple cost–control mechanism, it has limited effect on health-care providers and may restrict access to health care as patients will need to make an out-of-pocket payment for part of the health-care cost. Furthermore, wrong selection of people and services to be covered may limit the long-term financial sustainability of a health insurance scheme that is disproportionately composed of sick people, and with little or no enrolment of people who are healthier because of the high premiums that they must pay.

Advances in information and communications technologies and data analytics, if appropriately applied, may better enable health systems to address these concerns, and optimize the design of health insurance policies. A large amount of data has become available in different forms, and with varying levels of accuracy and reliability in what has become known as the big data phenomenon.^13^ The big data phenomenon broadly relates to a social phenomenon characterized by the availability of very large amounts of highly varied data from many different sources that may be processed rapidly. This phenomenon has contributed to the development of new data analytics and the use of sophisticated technologies, such as artificial intelligence,^14^ to combine, process and analyse these aggregated data to make predictions, and support optimal decision-making based on these predictions. In the context of health insurance, different algorithms have been developed to predict future costs, and the 10 most widely used of these algorithms have been identified by the Society of Actuaries.^15^ Algorithms to predict costs have been used not only by private insurers, but also by not-for-profit hospitals, academic groups and governmental agencies.^16^^–^^18^

Here we discuss the ethical and legal implications of a growing interest among health insurers to complement traditional methods of cost prediction with such big data and data science analytics.^19^^,^^20^ The private sector has led the development of big data analytics based on artificial intelligence in response to several concerns, especially those concerns relating to risk of loss from large claims being made and fraud detection.^21^ Insurance risk-scoring algorithms are developed to predict the likely loss ratio relativity of an individual,^22^ that is, whether the cost of that individual’s insurance claims relative to premiums paid will be higher or lower than the average. Advantages of the use of such scoring algorithms include: reliability, because improved capability in forecasting the future health status of insured people could be more accurately related to the risk of loss; and efficiency, from more precise allocation of resources.^23^ Typically, such scores are not used in isolation to set premium prices or to determine insurability, i.e. whether a particular person can be insured. Most insurers use an insurance score together with other kinds of information (e.g. medical claims history) to support evaluations. While big data analytics have enabled the development of new rating factors, these new factors have led to a larger number of smaller risk pools, i.e. specialized health insurance programmes for people who are not able to obtain regular insurance coverage because of costly pre-existing conditions. Where interconnectedness of these risk pools is limited, bias arising from the limited number of data sets could penalize already vulnerable individuals.^24^ There is currently no evidence that an increasing level of detail of risk assessments has led to high-risk individuals being excluded from coverage.^25^ However, a widely used algorithm has been shown to have significant racial bias because black patients in the United States of America are depicted as much sicker than white patients at a given risk score.^16^ In contrast, a highly interconnected and integrated data system could lead to concerns about an intrusive society, where individuals are closely monitored by data controllers.^26^

The use of big data and data analytics in health insurance have potential benefits, and may even be necessary to overcome persistent challenges to effective and equitable risk sharing, and to strengthen health systems. However, an ethically sound and enabling environment needs to be established and sustained to support, and perhaps even steer, such technological development and implementation in health insurance. In the next section, we argue that building and maintaining trust and trustworthiness is a central feature of an appropriate ethical environment. We then consider safeguards, policies, and participatory and collaborative mechanisms that could be introduced to promote trust and trustworthiness.

## Enabling an ethical environment

The ethical relationship between insurer (whether public or private) and the insured person is based on trust.^27^ Interpreting this ethical relationship from a legal standpoint, trust is also a means of overcoming the problem of asymmetric information, i.e. a situation where transacting parties do not have equal access to relevant information.^27^ The insured person is expected, and legally required in many countries, to act in good faith, and the insurer is also legally required to conduct itself with integrity. Trust underscores data governance policies, which seek to give insured people greater control over their personal data and specify limits on data that insurers may collect for actuarial purposes, that is, where insurers make statistical calculations of health status and/or life expectancy to determine the insurability of a person or the premium payable. For example, certain types of genetic information may not be used by insurers to decide on insurability in some countries. The ethical and legal language of privacy is a common expression of such control in the context of health and health-related matters. Research on guidelines on data sharing issued over the past two decades identified autonomy and privacy of people, and the quality and management of their data as the three most common themes.^28^ Privacy was also found to be an all-encompassing and overarching issue related to the disclosure of personal information.^29^ In addition, the degree of control over one’s personal information and trust were also identified as ethical concerns that arise from the use of personal data collected in a person’s life.^29^ With the growing use of big data analytics, is information control through privacy and related arrangements sufficient to sustain trust?

In its first intergovernmental standards on artificial intelligence to sustain trust and trustworthiness, the Organisation for Economic Co-operation and Development called for a more diverse ethics and governance framework to be developed.^30^ All actors with an active role in artificial intelligence systems, including organizations and individuals that deploy or operate artificial intelligence, are called to promote and implement five complementary value-based principles for the responsible stewardship of trustworthy artificial intelligence. These principles are: (i) inclusive growth, sustainable development and well-being; (ii) human-centred values and fairness; (iii) transparency and explainability (i.e. that processes are evident and interpretable); (iv) robustness, security and safety; and (v) accountability. In addition, five recommendations are directed to policy-makers, which include shaping an enabling policy environment for trustworthy artificial intelligence. An independent expert group of the European Commission responded with an ethical framework, based on human rights and rooted in respect for human dignity.^31^ The four principles that are put forward in the framework, respect for human autonomy, prevention of harm, fairness and explainability, argue for a human-centred approach to building and sustaining trust.

The ethical requirement of explainability is important, because a big data analysis based on artificial intelligence may be unable to generate an explanation of its decision. Since the analysis relies on many of single associations, identifying the technical and logical reasoning behind a decision made by such an algorithm may be difficult. In a health-care context, it is currently difficult, if not impossible, to say if a computer-aided diagnostic based on artificial intelligence is more likely or not to detect a rare disease condition compared with a clinical expert.^32^ Others have argued that big data analytics based on artificial intelligence are not necessarily unaccountable black-box software systems where decisions cannot be explained. The problem of non-transparent decision-making may be avoided by requiring, through effective governance, rigorous science and engineering in system design, operation and evaluation.^33^^,^^34^ The use of big data analytics, whether in health insurance or another context, is trustworthy if the outcome can be validated by the end users and/or governance bodies. Arguably, a lack of explainability and possibly even bias already exist in the ways that cost predictions and actuarial risk assessments are applied in health insurance and other settings.^35^ From a sociotechnical standpoint, such lack of explainability reflects information and power imbalances between the software developers on the one hand and users on the other. If left unchecked, big data analytics based on artificial intelligence will widen existing power differences, which will in turn compromise trustworthiness and generate mistrust.

## Ensuring ethical standards and trust

Big data analytics based on artificial intelligence can potentially enable health systems to deliver better quality, and more personalized and responsive products and services, possibly a form of intertemporal insurance whereby premiums payable better reflect current health status.^36^ However, regulatory safeguards, policy guidance and operational mechanisms are required to ensure that such analytics are applied for health insurance purposes in ways that meet ethical standards and are trustworthy. While the exact nature and form of safeguards, policies and mechanisms will depend on the specific context and conditions to which they relate, we describe four examples of such measures.

### Data governance framework

A clear and effective data governance framework is essential. In Europe, the General Data Protection Regulation seeks to secure the autonomy, dignity and privacy of people in terms of specific rights.^37^ Of these rights, the rights relating to automated decision-making (i.e. making decisions solely using automated computer programs without any human involvement), including profiling of an individual based on personal data, are the most relevant to our paper. The regulation states that automated decision-making that has legal or similarly significant effects is permissible only if this decision-making is necessary for entry into or performance of a contract, authorized by law, that is applicable to the data controller, or is based on the explicit consent of the person whose data are being used. In addition, data controllers must carry out regular checks to ensure that the system is working as intended, inform people affected about the processing and provide simple ways for them to challenge a decision. For instance, analytics based on artificial intelligence are used to analyse health insurance claims to detect fraud. Denial of claims based solely on algorithms will not be permissible under the European regulation. Arguably, this current legal safety net is only a baseline, and data controllers and processors will need to do more to meet the standards of the human-centred approach recommended by the European Commission report on trustworthy artificial intelligence.^31^

### Transparent rules

A clear process is necessary to determine what and how much information should be considered for the purposes of health insurance to guard against unfair discrimination. In the USA, for instance, discrimination by a public insurer based on race, religion or nationality, may be prohibited by law regardless of actuarial justification. This actuarial justification is the statistical relationship established between a particular characteristic of the insured person or an environmental condition and the designated outcome, be it claim frequency, claim severity, pure premium (i.e. the fraction of the premium payment that is used to pay the probable losses), loss ratio (i.e. the proportion of the claims paid by an insurer relative to the premiums earned) or fraudulent claim. Under United States constitutional law, the use of traits, such as race to classify particular groups of people is subject to higher legal inspection, called suspect classification in American jurisprudence. ^38^ This classification is legally called suspect because a history of discrimination on the basis of race exists, race is an immutable characteristic that bears no relationship to a person’s ability to contribute to society, and the racial group concerned may lack political power.^38^ For this reason, the Affordable Care Act states that insurance companies can take account of only five factors when setting premiums: age, location, tobacco use, individual versus family enrolment, and proportions of contribution by the insurer and the insured.^39^ Predictive models identify attributes that are correlated with loss to the insurer, some of which may also correlate with race. In this respect, differences in insurance premiums may reflect broader social inequality, and perpetuate historical biases not only of the data, but also of the software developers. If insurance coverage is required by law and people are penalized for not maintaining the insurance, then it is necessary for insurance regulators to monitor the availability, affordability and actual health outcomes, particularly where individuals who are likely to suffer from unfair discrimination are concerned.

### Participatory mechanisms

Big data could help improve understanding of human interactions, and provide opportunities to involve people in how their personal data should be managed and governed.^40^ Through suitably constructed participatory mechanisms, people can decide on how their personal data may be consolidated from different sources and made available to insurers under conditions that promote trust. These mechanisms help to reduce biases in data and analytics modellers, and improve public confidence in fair treatment by insurers. The rationale behind a more participatory arrangement is much the same as for privacy (and data protection) in the General Data Protection Regulation, and is consistent with the human-centred approach outlined in the report of the European Commission on trustworthy artificial intelligence.^31^ Empowering people in this way can better enable the implementation of a value-based insurance design to improve health-care quality and decrease costs. A value-based insurance design attempts to promote access to, and use of, high-value clinical services.^41^ For example, by fully covering or lowering cost sharing of preventive services or medicines, for example medications to control blood pressure or to treat diabetes, such an insurance plan can lower total programme costs by reducing future expensive medical interventions. Unlike conventional insurance designs, cost sharing in a value-based insurance design is not based on the cost of providing a specific service or product, but on matching cost sharing with its clinical value. For this reason, people covered under value-based insurance will pay a lower price for high-value services through lower out-of-pocket payments and a higher price for lower-value services, for example, head imaging for uncomplicated headache.^42^^–^^44^

### Collaborative governance

Regulation of big data analytics is increasingly decentralized, varied and, in the health-care context, collaborative.^45^ A platform should be established to enable insurers and governance bodies, including regulators and policy-makers, to work together to make big data analytics as transparent and accurate as possible. Even if not intended, unfair discrimination can arise if price differences do not reflect the difference in expected loss and expenses. This situation may happen because of defects in analytical models, selection bias, inaccurate data, or data that incorporate subjective judgements, among other issues. In the era of big data, it is important to be aware that quantity of data does not mean that concerns about quality and reliability of conventional data collection and analysis no longer matter.^46^ Efforts to increase interoperability in health-data infrastructures remain challenging in many countries. These efforts may have even been made more complex as health-financing systems become increasingly combined, with different public and private payers and providers, and with many combinations in between. Meeting these challenges will require the relationship between insurers and regulators to be reconceptualized. 

## Conclusion

Big data technologies could potentially enable insurers to: improve sustainability reporting (as a mechanism to study the overall performance and impact of the organization’s work and activities); create new tools for loss mitigation; and allow them to gain deeper understanding of factors related to health insurance design from their data sources. However, inappropriate design and application of such technologies will compromise trust and trustworthiness. The current insurance governance framework does not adequately equip interested stakeholders to respond to anticipated ethical and legal concerns.^47^ Current insurance governance also fails to give insurers incentives to be more responsive to the needs of insured people or to apply big data analytics in ways that are trustworthy. Regulators and policy-makers must recognize that market forces alone cannot protect public interests or secure trustworthiness. Insurers must similarly recognize that compromising trust and trustworthiness will ultimately damage their long-term interests and sustainability. To harness the benefits of big data, the insurer–regulator relationship should be increasingly collaborative and the insurer–insured relationship should be increasingly participatory.

The World Health Organization recently published its guideline on the development of digital health interventions for health system improvements.^48^ Although these recommendations are mostly directed at specific categories of digital health interventions, such as digital tracking of the health status of a client and related services within a health record, some of the considerations on digital health interventions are applicable to the discussions in this paper. Perhaps of greatest relevance is the explicit recognition that technology is not a substitute for well-functioning health systems, and that there are significant limitations to what technology is able to resolve. Advanced computing sciences in big data and artificial intelligence, whether applied in a health or health-insurance context, can enhance health systems and support the realization of UHC. However, such technology will not replace the fundamental components of the health system, which include the health workforce, and leadership and governance. Just as digital health interventions will need a robust enabling environment to be effective and beneficial, such an environment is also necessary for the development and application of big data analytics in health insurance. Unless the necessary safeguards, public policy guides and participatory mechanisms are in place, big data analytics will very likely worsen the proliferation of unconnected systems and existing inequities, as well as erode trustworthiness and trust.
